# Carbon Nanotubes for Improved Performances of Endodontic Sealer

**DOI:** 10.3390/ma14154248

**Published:** 2021-07-29

**Authors:** Andreea Marica, Luminita Fritea, Florin Banica, Cosmin Sinescu, Ciprian Iovan, Iosif Hulka, Gerlinde Rusu, Simona Cavalu

**Affiliations:** 1 Faculty of Medicine and Pharmacy, University of Oradea, P-ta 1 Decembrie 10, 410087 Oradea, Romania; andreeamarica94@yahoo.com (A.M.); dr.iovan@biostandard.ro (C.I.); Simona.cavalu@gmail.com (S.C.); 2 Faculty of Dentistry, “Victor Babes” University of Medicine and Pharmacy, P-ta E. Murgu 2, 300041 Timisoara, Romania; minosinescu@yahoo.com; 3 Research Institute for Renewable Energies, University Politehnica Timișoara, G. Muzicescu 138, 300501 Timișoara, Romania; iosif.hulka@upt.ro; 4 Faculty of Industrial Chemistry and Environmental Engineering, University Politehnica Timișoara, C. Telbisz 6, 300001 Timișoara, Romania; gerlinde.rusu@upt.ro

**Keywords:** endodontic sealer, CNTs, chlorhexidine, silver nanoparticles, antimicrobial

## Abstract

In order to overcome the limitations of current endodontic sealers, especially against resistant bacteria, recent developments in the field of nanotechnology have proved the necessity to reconsider the composition and physico-chemical properties of classical sealers. Nanoparticles with their unique features in terms of small size and high specific surface area, are the best choice for incorporation of antiseptic agents and effective delivery. The aim of our study is to prepare a novel platform for antibacterial drug delivery in dental adhesive systems used in endodontics. For this purpose, multi-walled carbon nanotubes (MWCNTs) encapsulating chlorhexidine (CHX) and colloidal silver nanoparticles (AgNPs) were prepared and incorporated into commercial sealer and investigated in terms of bonding performance to dentin and effectiveness against *E. faecalis*, *S. aureus* and *Candida albicans*, which are responsible for the majority of the failures in endodontic treatments. In this context, the challenges related to the long-term biological effects of CHX/AgNPs loaded MWCNTs are discussed.

## 1. Introduction

The development of “smart” endodontic therapeutic agents and materials has emerged in the context of a new era of nanomaterials. Despite the reported success rate of classical endodontic treatments (96%) [1,2], failure still occurs as a result of improperly cleaning and shaping of root canal, or microleakage of sealing material [3]. A combination of adequate instrumentation, irrigation, root canal obturation and sealing are the essential steps for successful endodontic treatment. The main goal of the treatment is to eliminate the microorganisms and to prevent root canal reinfection, knowing that the primary causes of apical periodontitis are related to the microbial infection of the pulp, produced by metabolic products [4]. However, due to the complexity of anatomical and morphological architecture of root canal system and the resistant bacteria, the complete cleaning by chemo-mechanical techniques cannot be achieved. The traditional root canal irrigants are hypochlorite (NaOCl) and chlorhexidine (CHX), which are extensively used in endodontic therapy with good antimicrobial efficacy against both Gram-positive and Gram-negative bacteria and acceptable biocompatibility [5,6,7,8]. There are some limitations, however, as the direct application of NaOCl is associated with tissue destructions (mainly dissolution of collagen network, due to the braking of carbon bonds, affecting the primary structure), depending on the concentration and application time [6]. On the other hand, chlorhexidine digluconate showed a dose dependent inhibition mechanism and positive effect in stabilization of resin dentinal bonds when was applied after acid etching [9,10]. The final stage of non-surgical root canal treatment consists of application of endodontic sealers, a tree dimensionally obturating root canal system, acting for long term and being capable of filling the voids between gutta-percha and dentinal canal walls [11,12]. The adequate filling of the empty space is crucial in completing the sterility, and hence, preventing reinfection of root canal. According to the literature [12,13,14,15], the requirements of an ideal root canal sealer are: (1) excellent sealing ability when set; (2) sufficient setting time to ensure working time; (3) dimensional stability; (4) insolubility against tissue fluids; (4) good adhesion to canal walls; (5) suitable antimicrobial properties; and (6) biocompatibility.

A great variety of endodontic sealers are commercially available and classified according to the chemical composition: zinc oxide eugenol, epoxy resin-based, glass ionomer-based, calcium hydroxide-based and methacrylate-resin–based sealers [15,16,17]. In terms of biocompatibility, previous studies suggested that bioactive sealers may exhibit lower cytotoxic potential compared to other types of root canal sealer [17].

Nanotechnology has lately emerged as a discipline at the interface of chemistry and life sciences; nanoparticles and carbon-based nanomaterials have attracted huge interest in the recent years being widely applied in the biomedical field [18,19,20,21,22,23]. The concentrated efforts to develop “nanomodified” dental materials resulted in the incorporation of various nanoparticles to increase the surface area between the dentin and the obturating material. The smaller particles size increases the contact surface area and hence, possesses a higher antimicrobial effect than the macro sized material [24,25,26]. 

Since nanoparticles are able to penetrate into dentinal tubuli, the development of new generation of endodontic sealers is an important goal of improving comprehensive oral health. In the field of endodontics, the development of nanomaterials is focused toward overcoming the microbial challenge, and hence, new sealers are expected to possess multiple actions in terms of better sealing capacity, remineralization and enhanced antimicrobial effect [26,27,28,29]. Especially AgNPs are well known to interact with the bacterial cell membrane, and consequently, to increase permeability and prevent DNA replication. However, the increased cytotoxicity of AgNPs at elevated concentrations, due to their size and surface characteristics, may cause nonspecific oxidative damages [30,31]. Examples of organic and inorganic antimicrobial nanoparticles used in root canal obturation and adhesives are: silver nanoparticles (AgNPs), copper CuNPs, ZnO NPs, hydroxyapatite NPs, TiO_2_ NPs, chitosan NPs and quaternary ammonium polyethyleneimine NPs (QAPEI), as presented in a recent review paper by Makvandi et al. [27]. Among the organic nanostructures, carbon nanotubes (CNT) have unique physico-chemical, mechanical and electrical characteristics, such as high stiffness, axial strength and excellent thermal conductivity, due to their cylindrical graphitic structure, with nano-sized diameter and larger aspect ratio [1,2]. The Young modulus value is much higher than those of typically found in stainless steel and carbon fibers [2]. CNTs also have the capacity to easily pass the biological barriers and the ability to carry macromolecules unable to pass through cellular membrane by themselves, leading to novel, biocompatible delivery systems, including in endodontic treatments [2,28]. 

The majority of antibiotics and antiseptic agents provide short-term antibacterial effects, with a risk of developing antibacterial resistance. In this context, the aim of our study was to prepare a platform for long-lasting antibacterial and antifungal drug delivery, as a novel endodontic sealer obtained by nanomodification, incorporating a mixture of CNTs, CHX and AgNPs. The possible synergic effect of AgNPs, CHX 2% and CNTs was also explored, without altering the structural, ultrastructural and thermal behavior of the original sealer. The phyisico-chemical, structural and morphological characterization of both the original and nanomodified sealer was performed by using FTIR spectroscopy, scanning electron microscopy (SEM), cyclic voltammetry and nanoindentation measurements, with focus on the assessment of interfacial adaptation to root canal dentine. The impact of CNTs loaded with CHX/AgNPs mixture on the nanomechanical properties of the sealer was also discussed. Complementary, thermal analysis (TGA) were performed in order to investigate the stability of modified sealer upon addition of CNTs/CHX/AgNPs mixture. The antimicrobial and antifungal efficacy of the novel endodontic sealer was tested against *Enterococcus faecalis*, *Staphylococcus aureus* and *Candida albicans*. 

## 2. Materials and Methods

### 2.1. Preparation of CHX/AgNPs Loaded CNTs and Incorporation into Endodontic Sealer

The starting materials were commercial clorhexidine gluconate gel GLUCO-CheX 2% (Cerkamed, Stalowa Wola, Poland), ionic colloidal silver Argentum Special Pure Life^®^ 77 ppm (Agnes Itara SRL, Suceava, Romania), multi-walled CNTs (NC3100, Nanocyl, Sambreville, Belgium) and ADSEAL (resin-based root canal sealer, META^®^ BIOMED, Chungcheongbuk-do, Korea). According to the manufacturer, the formulations consist of ionic silver with purity 99.99% and particle size 4–9 nm (determined by TEM), while the size of the CNTs are 9.5 nm in diameter and 1.5 µm length. The composition of ADSEAL consists of: base (epoxy oligomer resin, ethylene glycol salicylate, calcium phosphate, zirconium oxide, bismuth subcarbonate) and catalyst (poly butanediol aminobenzoate, triethanolamine, calcium phosphate, bismuth subcarbonate, zirconium oxide).

The modified sealer was prepared as follows: 10 mL of colloidal silver (77 ppm), 12.5 mg CNTs and 0.5 mL CHX 2% were mixed by sonication and stirring during 1 h. A volume of 1 mL of this mixture was left overnight for water evaporation (leading to a gel composition) and then was added to 1 g of commercial endodontic sealer, and mixed until homogenized, following the manufacturers’ instructions. The composition was applied on the endodontic canal of 20 human permanent incisors, specially prepared, as described in the Section 2.7. 

### 2.2. FTIR Spectroscopy

The structural characterization of both commercial and nanomodified endodontic sealer was performed using an FTIR 780 spectrophotometer (PG Instruments, Leicestershire, UK) and the KBr technique, operating in the range of 400–4000 cm^−1^, with a scanning speed of 32 cm^−1^ and spectral width of 2.0 cm^−1^. For this purpose, after hardening, the specimens were ground using a dental milling machine (Benco Dental, Pittston, PA, USA) in order to obtain a fine powder suitable for KBr pellet preparation. FTIR spectra of CNTs as supplied from the manufacturer, were also recorded in the same conditions. CaF_2_ cell accessory was used in order to acquire the FTIR spectra of colloidal mixtures containing CNTs, CHX and AgNPs.

### 2.3. SEM Examination (Mixture AgNPs/CHX/CNTs, Commercial and Nanomodified Sealer)

For SEM investigation, the materials were prepared according to the manufacturer instructions and applied on specially designed plastic inserts and left until hardening. The microscopic details were recorded on fracture surface of both commercial and modified material using a Leo 438VP electron microscope (SEM, Oberkochen, Germany), operating at 30 kV, with a variable vacuum level. In order to study the detailed morphology, structure and elemental composition, the polished specimens were characterized by a Quanta FEG 250 SEM instrument (FEI, Breda, The Netherlands) using a back scattered electron detector (BSD) and energy dispersive X-ray spectroscopy (EDX, using an Apolo SSD detector, EDAX Inc., Mahwah, NJ, USA). The microstructure investigations and EDX analysis were performed at about 10 mm working distance (WD) in low vacuum mode in order to avoid surface charging and damage to the analyzed samples. The details of the CNTs before and after loading with AgNPs/CHX mixture were also recorded in the same conditions. 

### 2.4. Electrochemical Measurements

Screen printed electrodes (DRP 110, Metrohm-DropSens, Oviedo, Spain) were used for electrochemical analysis. The setup consisted of a Ag reference electrode, carbon counter electrode and another carbon electrode as a working one. The experiments were carried out using a potentiostat Autolab PGSTAT 128N (Metrohm, Utrecht, The Netherlands) equipped with Nova 2.1.2 software. In order to assess the electrochemical behavior of the colloidal mixture and of its individual components, differential pulse voltammetry (DPV) was performed employing the following parameters: start potential −1 V, end potential +1.5 V, step 0.01 V, modulation amplitude 0.05 V, modulation time 0.05 s, interval time 0.1 s. In the case of ionic Ag solution, a reduction process was previously applied using the same DPV method.

### 2.5. Nanoindentation Measurements

The endodontic sealer specimens (with and without modifications) were subjected to nano-mechanical tests using a depth sensing Nanoindenter G200 device (Agilent Technologies, Santa Clara, CA, USA), at room temperature and normal humidity (45–52%), applying a diamond Berkovich pyramidal shaped tip. Each indentation test (consisting of a loading and unloading phase) was repeated 12 times, the microscopic image of the selected area on the sample surface allowed precise control between the sample position and the indenter. The values of the Young modulus were obtained from load–displacement curves, by fitting parameters, using the Oliver–Pharr method [29], taking into account the tip shape function and the fact that the measured displacement may include contributions from both the specimen and the indenter. 

### 2.6. Thermogravimetrical Analysis (TGA)

TGA thermograms were recorded using a TG 209 F1 Libra (NETZSCH-Gerätebau GmbH, Selb, Germany) thermogravimetric analyzer. The measurements were carried out in a nitrogen atmosphere, in the temperature range of 20–850 °C, with a heating rate of 10 °C/min. The data were processed with the Netzsch Proteus-Thermal Analysis program version 6.1.0. (NETZSCH-Geraetebau GmbH, Selb, Germany).

### 2.7. SEM Investigation of the Interfacial Adaptation to Root Canal Dentine

A total of 20 human permanent incisors were selected for this study. The selected teeth were extracted for orthodontic reasons, with patient informed consent, available from the Dental Clinic of Faculty of Medicine and Pharmacy, University of Oradea. The protocol was approved by the Institutional Research Ethics Committee of the Faculty of Medicine and Pharmacy, University of Oradea [Nr. 2/31.03.2021]. As a first step, the teeth were endodontically prepared, by making an access cavity with a round diamond bur. After the access cavity was made, each tooth was irrigated with 5% sodium hypochlorite (Cerkamed, Stalowa Wola, Poland). The next step was to determine the working length with a stainless-steel 0.10 K-file (VDW, Munich, Germany) and an endodontic ruler (Angelus, Londrina, Brazil). Having the working length determined, the next step was to enlarge the coronal one third of the canal with an endodontic preflaring file-SX file (0.19/0.04 taper) from the Protaper Universal system, at 250 rpm and 3.0 torque, with the endodontic motor X-smart plus (Dentsply Maillefer, Ballaigues, Switzerland). The glide path was established with Proglider File (0.16/0.02 variably, Dentsply Maillefer, Ballaigues, Switzerland). The next step was to prepare the teeth with the endodontic file system, ProTaper Universal (S1 0.17/0.02, S2 0.12/0.04, F1 0.12/0.07, F2 0.25/0.08, F3 0.30/0.09 Dentsply Maillefer, Ballagues, Switzerland) and cleaning with 5.25% NaOCl. A final rinse was made with 17% EDTA solution (Cerkamed, Stalowa Wola, Poland), followed by a 10 min ultrasonic treatment in distilled water. A longitudinal section was applied to each specimen using a diamond coated disk, in order to expose the root canal. Half of the sections of the prepared teeth were coated, in the root canal area, with the commercial sealer ADSEAL, respectively, the other half with the modified one, according to the manufacturer recommendations and stored at 37 °C and 100% humidity for 48 h before SEM investigation. Each half was transversely sectioned in the median zone in order to investigate the ultrastructural details of the interfacial area between the sealer and dentine. In this way, it was possible to evaluate each individual specimen in terms of interfacial adaptation toward modified and unmodified sealer, allowing a fair comparison, taking into account the structural and morphological particularities of each root canal. The SEM images at the interface between the root canal and the sealer were recorded in back-scattering mode.

### 2.8. Antimicrobial and Antifungal Efficacy

The following strains were considered for antimicrobial and antifungal tests: 

Enterococcus faecalis ATCC^®^ 29212, Staphylococcus aureus ATCC^®^ 25923) and fungi (Candida albicans ATCC^®^ 10231), being supplied by Biostandard Laboratories Oradea. Five test tubes were prepared as follows: 1 mL of bacterial inoculum suspension in concentration of 1.5 × 10^8^ CFU/mL, which corresponds to standard 0.5 McFarland (prepared in Mueller Hinton broth) was added to 1 mL of each tested combination: (1) CNTs/AgNPs/ CXH 2%; (2) CNTs/H_2_O; (3) CNTs /AgNPs; (4) CNTs /CXH 2%; (5) AgNPs; (6) CHX 2%. The test tubes were incubated for 1 h at 37 °C, and then the content was spread on the surface of the inoculated broth in Petri dishes, and incubated for 24 h at 37 °C. Similarly, an antifungal test was conducted, except that Sabouraud Dextrose agar (Oxoid, Thermo Fisher Scientific Inc., Milan, Italy) was used for the cultivation of *C. albicans* strain, in a concentration of 2.0 McFarland. All the tests were performed in triplicate and the CFU/mL was determined by manual reading.

The positive controls were well known antimicrobial and antifungal drugs, gentamicin and nystatin. Different dilutions of the control drugs were prepared in order to assess their minimum inhibition concentration and the following concentrations were considered effective: 25 μg/mL gentamicin for *S. aureus*, 100 μg/mL gentamicin for *E. faecalis* and 5 μg/mL nystatin for *C. albicans*. In order to assess the antimicrobial activity of the set sealers, both solid samples (with and without the combination CNTs/AgNPs/ CXH 2%), set and aged for 7 days, were ground in a dental mill until a fine powder was obtained and the agar diffusion test was performed in this case. A small hole of 6 mm diameter was prepared in the center of each Petri dish and filled immediately with equal quantities of the resulted powder from both modified and commercial sealer. After 24 h incubation, the diameter of the inhibition area was measured. The assay was performed in triplicate and expressed as mean value ± standard deviation.

## 3. Results

### 3.1. FTIR Spectroscopy

**Figure 1:**
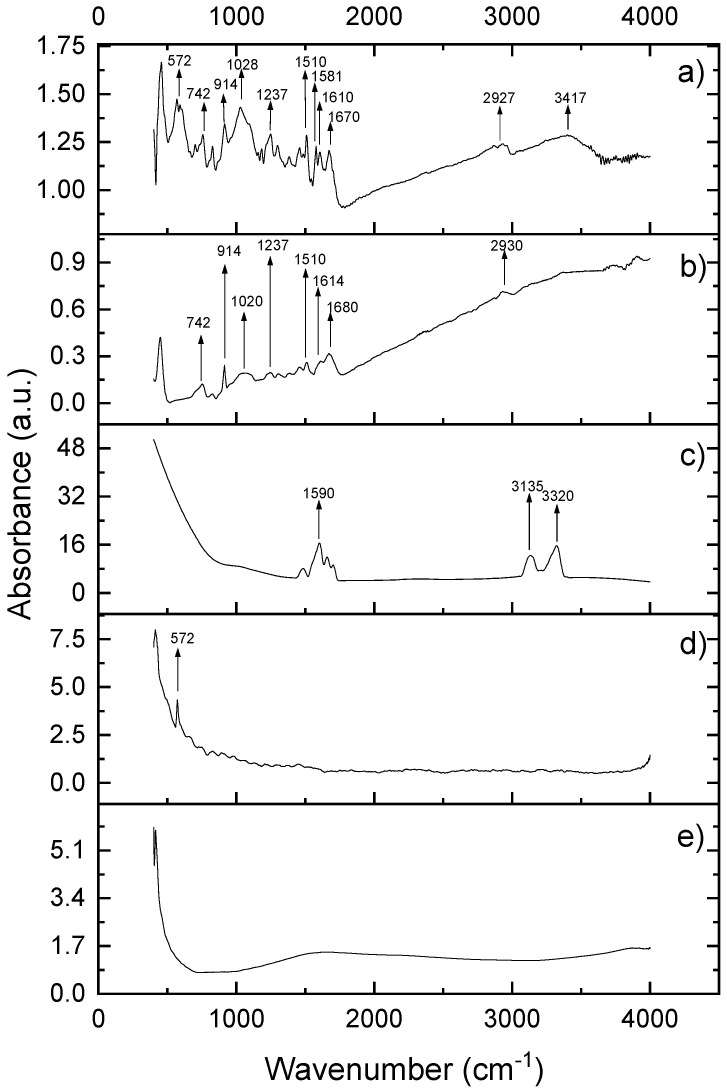
FTIR spectra of modified root canal sealer obtained by incorporation of (**a**) AgNPs/CHX/CNTs mixture; (**b**) commercial root canal sealer (ADSeal); (**c**) chlorhexidine gluconate gel; (**d**) colloidal AgNPs and (**e**) neat CNTs (as received from the manufacturer).

As presented in Figure 1, neat CNTs do not present any specific vibrational features, while AgNPs shows one single absorption peak at 572 cm^−1^, characteristic for Ag-O stretching vibration [30,31]. Chlorhexidine gluconate presents a characteristic, intensive peak at 1590 cm^−1^, accompanied by less intense satellites at 1650 and 1550 cm^−1^, which can be attributed to the C=C stretching of the aromatic moiety of CHX. In the high wavenumber regions, the two peaks at 3135 cm^−1^ and 3320 cm^−1^ regions are attributed to asymmetric and symmetric –NH vibrations, which are also suggestive of CHX. The main fingerprints of the commercial sealer are 742 cm^−1^ and 914 cm^−1^, which are due to the contribution of epoxy rings (the most important functional groups), 1020 and 1237 cm^−1^ corresponding to symmetrical and asymmetrical aromatic C-O stretch, 1510–1680 cm^−1^ corresponding to C-C stretching vibration in aromatic rings, and the peaks around 2930 cm^−1^ corresponding to symmetrical and asymmetrical C-H stretch of –CH_2_ groups [32,33,34].

The FTIR vibrational features of modified sealer preserved all the fingerprints of the blended components, while the main peaks of the epoxide groups are visible at the same wavenumbers as in the spectrum of neat sealer and the intensity of the bands at 572 cm^−1^, 1020 and 1237 cm^−1^ are enhanced. This behavior is due to the overlapping of C-O vibrations and those of the ether bonds. Additionally, in the high wavenumber region, the peaks between 2930–3417 cm^−1^ indicates the vibration of hydroxyl groups of epoxy resin superimposed over the asymmetric/symmetric –NH vibrations in CHX. As the main fingerprints of the neat sealer are preserved and no additional vibration bands (or disappearance of the existing ones) occurred, we can interpret that there is no chemical reaction between the original epoxy resin and the blended components (CNT, AgNPs and CHX). Hence, the sealer acts as a “reservoir” for the active ingredients.

### 3.2. Electrochemical Measurements 

**Figure 2:**
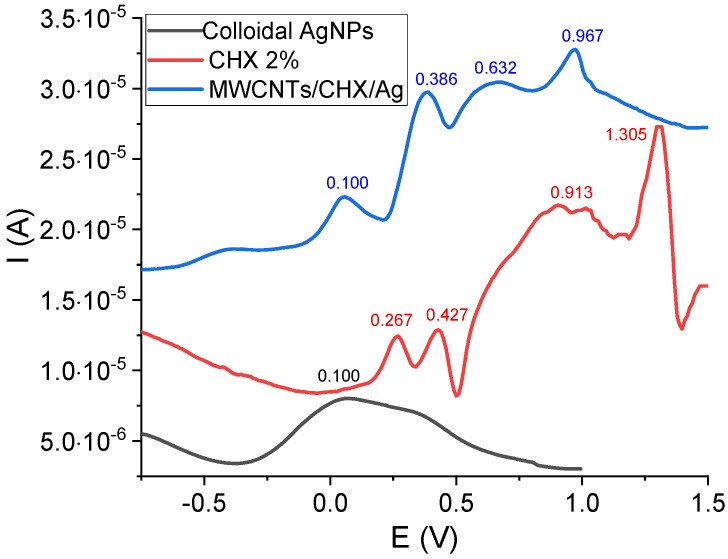
Differential pulse voltammetry of colloidal AgNPs, CHX 2% solution and the mixture CNTs/CHX/AgNPs.

The electrochemical profile of colloidal AgNPs, CHX 2% and the mixture CNTs/AgNPs/CHX 2% investigated on screen printed electrodes, is presented in Figure 2. The electrochemical oxidation of CHX revealed four anodic peaks at 0.267 V, 0.427 V, 0.913 V and 1.305 V. The ionic Ag^+^ solution indicated a cathodic peak at around −0.1 V (not visible in the figure) due to the reduction of Ag^+^, which was then re-oxidized at 0.1 V [35]. This oxidation peak was also found in the DPV of the mixture at almost the same potential. The main electrochemical features of CHX in the mixture CNTs/AgNPs/CHX2% were slightly shifted concerning the potential values (0.386 V, 0.632 V, and 0.967 V), while the first two oxidation peaks of CHX alone (at 0.267 V, 0.427 V) might be merged into one single peak in the case of the mixture (at 0.386 V). According to the literature [36,37,38], CHX oxidation leads to two p-chloraniline and two biguanidine molecules. 

### 3.3. SEM Investigation of Modified Root Canal Sealer 

**Figure 3:**
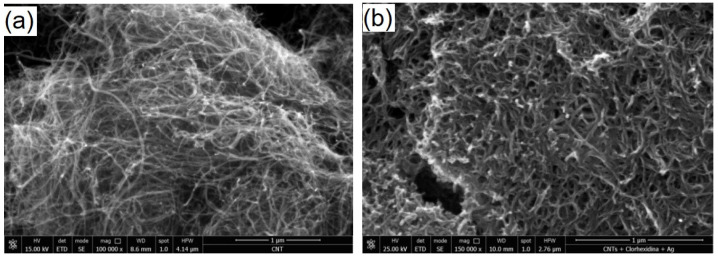

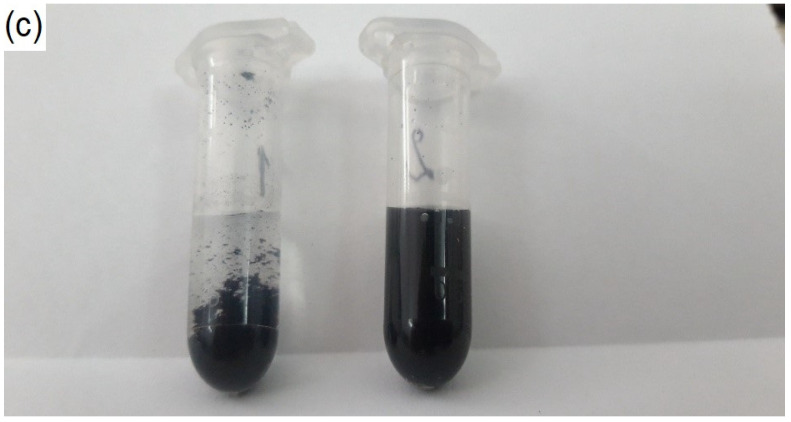
SEM morphological details of CNTs as received from the manufacturer, before (**a**) and after (**b**) loading with the mixture CHX/AgNPs (High magnification, 100,000×); (**c**) photographic image of colloidal CNTs in distilled water (left) compared to colloidal mixture CHX/AgNPs (right).

Prior to blending all the components, the morphology of CNTs was investigated (Figure 3). By comparing the morphological details of CNTs before and after loading with the mixture of CHX/AgNPs, an increased diameter of the nanotubes can be noticed after loading leading to a tighter matrix (Figure 3a,b). A good stability of CNTs loaded with CHX/AgNPs was also noticed compared to neat CNTs, when dispersed in distilled water, as presented in photographic images (Figure 3c), revealing the homogeneity of the colloidal mixture.

**Figure 4:**
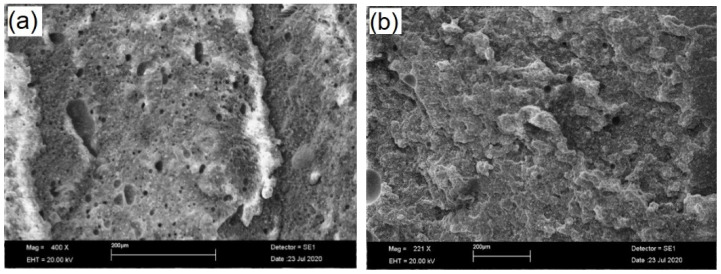
SEM images (low magnification, 400×) of the fracture surface of neat endodontic sealer (**a**) and modified sealer after incorporation of CNTs/CHX/AgNPs (**b**).

After blending the components and waiting for the setting time, according to the manufacturer’s instructions, SEM images were recorded on the fractured surface of neat and modified sealer, without any other surface preparation, as presented in Figure 4.

**Figure 5:**
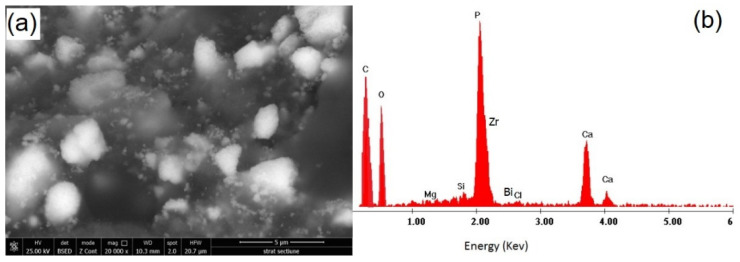

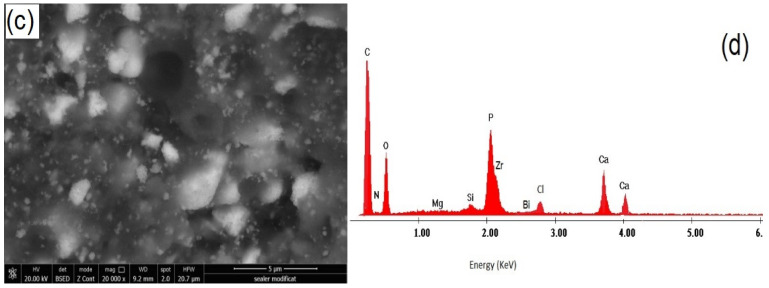
Ultrastructure details of commercial (**a**) and modified root canal sealer (**c**) along with the corresponding EDX spectrum (**b**,**d**).

It can be observed that there is a good dispersion of CNTs/CHX/AgNPs in the epoxy matrix, while multiple cleavage planes can be noticed on the fracture surface of the modified sealer, compared to the neat one. According to some authors [39,40,41,42,43] the better the nano-fillers disperse, the greater number and the smaller size of the cleavage planes are observed on the surface fracture of epoxy nanocomposites. Furthermore, the reinforcement mechanism is more effective when the height difference between the cleavage planes increases. High magnification details of the polished specimens are presented in Figure 5, along with the corresponding EDX spectrum. 

The details presented in Figure 5 emphasize not only a good dispersion of CNTs/CHX/AgNPs in the epoxy matrix, but also the presence of inorganic components and radiopacifiers—calcium phosphate, zirconium oxide and bismuth carbonate. The relative intensity of the main elements (especially C and O) is obviously influenced by addition of CNTs/CHX/AgNPs, although the presence of Ag was not identified in the EDX spectrum, due to the detection limit of the instrument.

### 3.4. Nanoindentation Measurements

**Figure 6:**
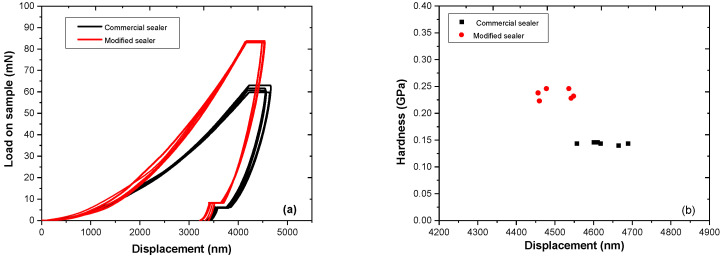
Load–displacement curves (**a**) recorded on the surface of commercial (black) and modified sealer (red) and the corresponding Young modulus calculation (**b**).

After setting and hardening, load–displacement curves were recorded for the two sets of prepared specimens, the commercial sealer and CNTs/CHX/AgNPs modified one, as presented in Figure 6a.

According to the curves’ profile, it can be observed that the addition of CNTs/CHX/AgNPs to the commercial epoxy-based sealer enhanced the nanomechanical properties of the composite: a peak force of 85 mN was necessary to apply on the surface of the modified sealer, compared to 60 mN for the commercial one, in order to reach the same displacement (3300 nm). Average elastic modulus values obtained from the fitting parameters revealed a maximum value E = 0.25 GPa for the modified sealer, compared with E = 0.15 GPa for the neat sealer. The results displayed in Figure 6 demonstrate a slight reinforcement due to the addition of CNTs/CHX/AgNPs, with respect to the selected concentrations, as described in Section 2.1. An increased trend of the modulus at higher CNT content has been reported previously [44,45].

### 3.5. Thermal Analysis

**Figure 7:**
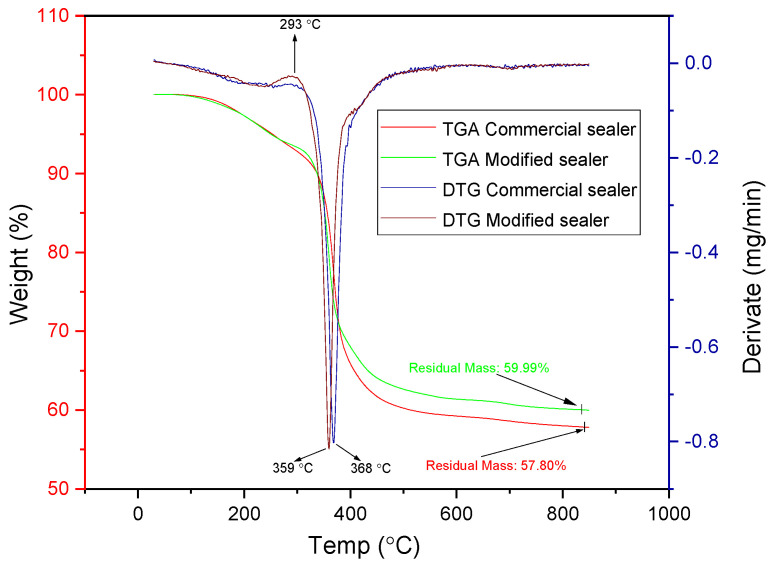
TGA and DTG thermograms of the modified and commercial sealers.

Thermogravimetric analysis (TGA) and first derivative curves (DTG) for the modified and commercial sealer are presented in Figure 7. It can be noticed that both sealers have almost the same degradation patterns, showing one single degradation step between 200 and 450 °C. In addition, there is no mass loss until 130 °C, which denotes the absence of water content in samples [43,44,45,46,47,48]. However, DTG curves indicates that there is a small difference between the decomposition point of modified and commercial sealer (359 °C and 368 °C), while the residual mass of the modified sealer is slightly higher (59.99% toward 57.80%), indicating a good stability upon addition of CNTs/CHX/AgNPs mixture. The small peak at 293 °C in the DTG curve indicates the thermal decomposition of AgNPs [43].

### 3.6. SEM Investigation of Interfacial Adaptation to Root Canal Dentine

**Figure 8:**
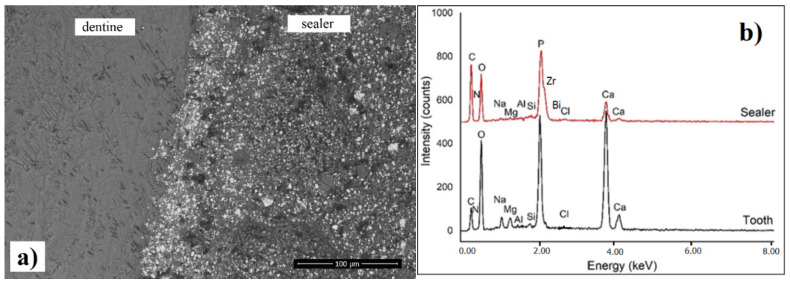

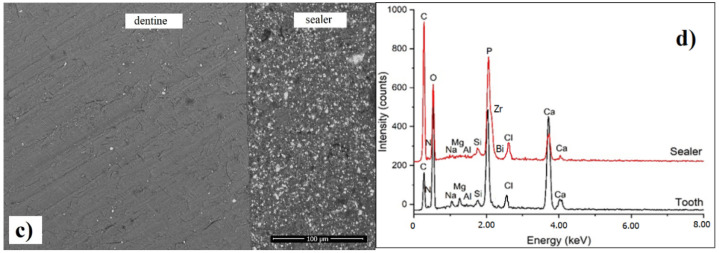
SEM images (**a**,**c**) of interfacial adaptation between sealer and root canal dentine (polished specimens) along with the corresponding EDX spectra (**b**,**d**): (**a**) neat sealer; (**c**) CNTs/CHX/AgNPs modified sealer. The transversal section was performed in the middle zone of the root.

It can be observed that there is a very good adaptation of both the commercial and modified sealers to the root canal walls, and no gaps were noticed across the investigated sections (Figure 8a,c). Moreover, based on the EDX spectrum recorded at the interface, it can be noticed that there is diffusion of chlorhexidine molecules from the sealer into the dentinal tissue, as evidenced by the presence of significant amount of chloride in the EDX spectrum (Figure 8b,d). We assume that AgNPs were also diffused across the interfacial area, but due to the instrument limitation, Ag was not visible in the quantitative spectrum. In addition, a more intense C peak was identified on the root canal dentine in contact with the modified sealer, suggesting the penetration of the modified resin component into dentine [44,49,50].

**Figure 9:**
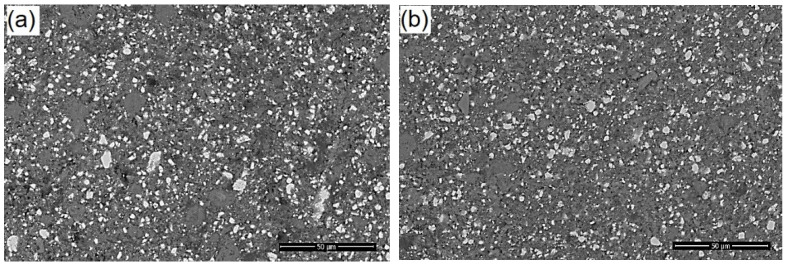
Back scatter SEM images with high magnification recorded on the surface of (**a**) neat sealer and (**b**) modified sealer, at the interface with root canal area.

The SEM details (back scatter mode) of the neat and modified sealer recorded at the interface are presented in Figure 9, emphasizing the details of inorganic compounds (white particles) and homogenous distribution within the resin matrix. Overall, it can be noticed that there is a similar microstructure of the modified sealer compared to the original one, suggesting that the addition of CNTs/CHX/AgNPs did not disturb the local distribution of the components, as there is no agglomeration of the calcium phosphate or radio opacifier particles related to the material.

### 3.7. Antimicrobial and Antifungal Effect of the Modified Sealer

**Figure 10:**
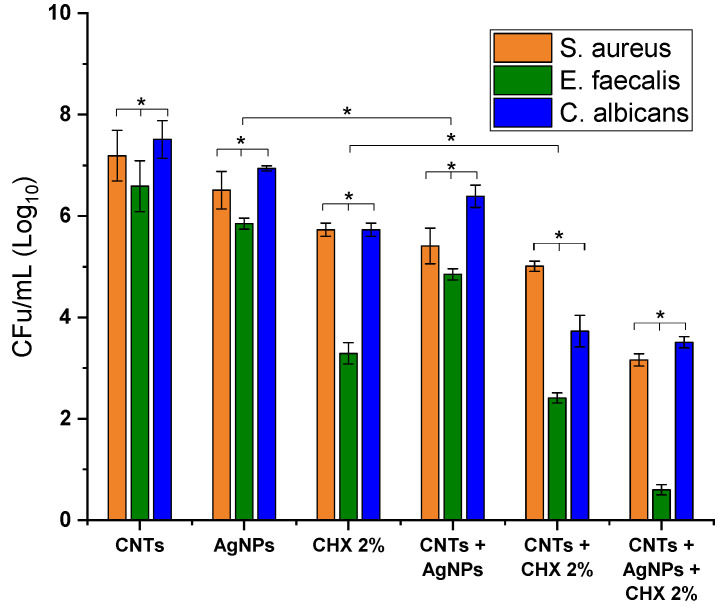
Antimicrobial and antifungal effect of different combinations and the mixture CNTs/AgNPs/CHX2% against the tested strains. Data are expressed as average value ± standard deviation of triplicate samples (statistical significance * *p* < 0.05).

In order to evaluate the antimicrobial effect of different combinations CNTs/AgNPs/CHX 2%, CNTs/CXH 2%, CNTs/AgNPs and their individual components, the prepared solutions were tested against *S. aureus*, *E. faecalis* and *C. albicans*, in concentration of 1 mg/mL. The results expressed as CFU/mL (Log_10_) are presented in Figure 10. The antimicrobial and antifungal assay clearly demonstrated the best efficiency of CNTs/AgNPs/CHX 2% mixture against *E. faecalis*. However, the efficiency of different combinations with respect to *E. faecalis* were noticed to follow the order: CNT + H_2_O < AgNPs < CNT/AgNPs < CHX 2% < CHX 2%/AgNPs < CNTs/AgNPs/CHX 2%. With respect to *S. aureus* and *C. albicans*, the effect was only moderate by comparison, however the same order being noticed. In addition, it can be observed that, if the active antimicrobial agent (AgNPs or CHX) was combined with CNTs, the effect was significantly enhanced, especially for the combination CNTs /CHX 2% against *E. faecalis* and *C. albicans*.

**Figure 11:**
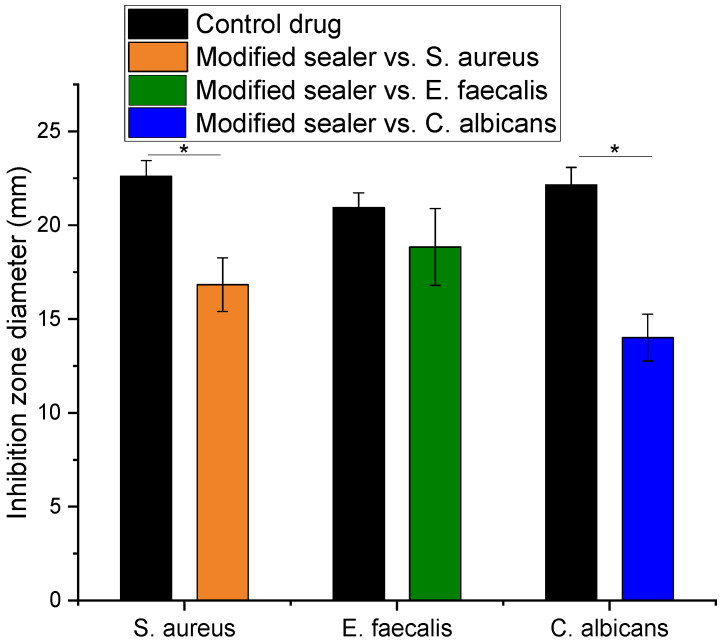
Diameter of inhibition zone in agar-diffusion assay with modified sealer and control drugs. Data are expressed as average value ± standard deviation of triplicate samples (* *p* < 0.05).

The agar-diffusion test applied for the commercial and modified sealer, 7 days after setting, revealed that the commercial sealer had no antimicrobial and antifungal effect. The measurement of the diameter of the inhibition zone was performed for the modified sealer with respect to each strain and presented in Figure 11, compared to the positive controls. In accordance with the previous assay, the maximum efficiency was noticed against *E. faecalis* followed by *S. aureus* and *C. albicans*. 

As compared to the controls, which are well known antibiotics and antifungal drugs, the results are considered very promising. For example, in the case of *E. faecalis* ATCC^®^ 29212, a concentration of 100 μg/mL gentamicin was found to effectively inhibit the bacterial growth, which is about six times higher than MIC reported for most of the isolated Enterococcus species [24,51]. In this case, no significant difference was noticed between the control and modified sealer (*p* < 0.05).

## 4. Discussions

The antibacterial ability of sealers is still insufficient and hence, continuous improvements are required, as the full impact of nanotechnology in endodontics is far from being fully understood. The incorporation of CNTs in epoxy-based matrix and the effective reinforcement is dependent on the aspect ratio, dispersion, alignment and interfacial stress transfer at the CNT-matrix interface [2]. It is generally considered that well-dispersed, randomly aligned CNTs are a preferred alternative for reinforcement, as aligned nanocomposites, in contrast to CNTs, possess anisotropic mechanical and electrical properties [52]. In addition to the reinforcement purpose, encouraging results have shown the ability of CNTs to be used as delivery systems for genes, peptides, oligonucleotides, cytotoxic drug molecules and antimicrobial agents [52,53,54]. Especially, the antimicrobial activity against Gram-positive and Gram-negative microorganisms, was evidenced due to the penetration of the sharp and narrow structure of CNTs through the cell’s membrane, concomitant with the drug release, compromising wall integrity and finally, death via cell lysis.

CHX is a well-known non-specific matrix metalloproteinase (MMPs) inhibitor, having a dose-dependent inhibition mechanism, based on the interaction with the sulfhydryl groups and cysteine site of MMPs [55,56]. Some previous studies attempted to incorporate CHX into etchant materials or into primers or adhesive components because it has been suggested that CHX effect is only available immediately after application of the modified materials [56]. AgNPs have been also incorporated into bonding agents or restorative materials in endodontics, attempting to reduce *E. faecalis* adherence to dentine, to eliminate biofilms, or as endodontic irrigants and intracanal drugs [8,31,57].

Gram-positive bacteria such as *S. aureus* and *E. faecalis* have been detected in periapical infections being responsible for endodontic failure [24]. Particularly, the incidence of *E. faecalis* was reported to be in the range from 24% to 77% in the cases of apical periodontitis [58], being also reported to be resistant to several antimicrobial agents. It has a great ability to invade the dentinal tubules, and, hence, to resist during the chemo-mechanical endodontic procedures. On the other hand, the occurrence of *C. albicans* in infected root canals varies between 1% and 17%, according to previous studies [59], being also resistant to conventional root canal irrigants. In this respect, the continuous improvement of endodontic sealers with high antimicrobial activity requires new developments with the aim to prevent or to decrease the bacterial growth within the root canal, while contributing to the repair of dentinal tissue. The currently available commercial endodontic sealers, possess different physico-chemical properties in terms of sealing ability, adhesiveness, solubility to oral fluids and dimensional stability [49]. As mentioned in literature, based on in vitro studies, different categories of sealers (such as resin, zinc oxide eugenol, calcium hydroxide, glass ionomer, silicon or silicate- based sealers) demonstrated good antimicrobial activity as freshly prepared, but the antimicrobial activity was lost as the material set [59,60,61,62]. Actually, no bacterial growth inhibition was noticed for 2–7 days set samples, as evidenced in a systematic review conducted by Al Shawaimi et al. [60]. However, the in vitro studies have some limitations due to the lack of standardization in terms of inoculum density, culture medium selection, agar viscosity, condition of plate storage, or diffusion–solubility relation of the material and culture medium. 

Our original approach, in the context of new generation sealers expecting to have a long-lasting antimicrobial effect, was to demonstrate that the antimicrobial effect of the mixture CNTs/AgNPs/CXH 2% incorporated in commercial sealer, was preserved long enough to efficiently inhibit Gram-positive germs, with excellent results towards *E. faecalis* in a concentration of 1 mg/mL. It is considered that epoxy resin-based root canal sealer possesses a certain degree of antibacterial properties attributed to bisphenol diglycidyl ether and formaldehyde released during the polymerization reaction [63]. Our results suggest that the modified sealer is able to release the antimicrobial agents long enough after setting and aging (7 days), which is an obvious improvement compared to the neat sealer. 

The structural properties of the modified sealer, evidenced by FTIR spectroscopy, suggests that no chemical reaction between the original epoxy resin and the blended components (CNT, AgNPs and CHX) was established during the preparation. Hence, the sealer acts as a “reservoir” for the active ingredients. According to the literature, CNTs interact with organic and charged molecules via π-π* and electrostatic interactions, while the small diameter of CNTs restricts the adsorption of larger organic molecules. Instead, most of the organic molecules can easily penetrate into the spaces between the tube bundles or at the external surface [2], being released in contact with dentine. The electrochemical profile of colloidal AgNPs, CHX 2% and the mixture CNTs/AgNPs/CHX 2% suggest that electrons transfer involving Ag^+^ and CHX molecules might be responsible for the synergic antimicrobial effect. 

A good dispersion of CNTs/CHX/AgNP in the epoxy matrix was demonstrated by SEM investigation and a slight reinforcement was noticed compared to the commercial sealer, as evidenced by nanoindentation measurements, which is in agreement with previous reported data [45]. The addition of CNTs/CHX/AgNPs did not disturb the local distribution of the inorganic components, as no agglomeration of the calcium phosphate or radio opacifier particles was noticed. 

In terms of thermal behavior, both modified and commercial sealers have almost the same degradation patterns, while only a small difference in residual mass was noticed. Overall, a good stability of the modified sealer can be noticed. The corroborated results clearly demonstrated that the modifications induced by addition of CNTs/AgNPs/CHX 2% mixture did not impair the structural, ultrastructural and thermal behavior of the original sealer.

Moreover, a similar, very good adaptation, without gaps, of both commercial and modified sealer to the root canal walls was evidenced by SEM. The EDX spectrum recorded at the interface, demonstrated the diffusion of chlorhexidine molecules from the sealer into the dentinal tissue. It is well known that chemical or mechanical dentin pretreatments (for example sandblasting procedure) are usually made in order to increase the roughness of the treated surface, which influences the adhesion properties and the contact area between the dentinal tissue and the sealer [64]. In our work, we did not apply any mechanical pretreatment, except the routine procedure of endodontic cleaning.

The antibacterial and antifungal assay clearly demonstrated a synergic effect of AgNPs, CHX 2% and CNTs with excellent results towards *E. faecalis*, which is responsible for the primary etiologic factors in pulp and periapical lesions [7].

## 5. Conclusions

In this work, we report the improved antibacterial effect of a modified endodontic sealer, upon addition of CNTs incorporating CHX 2% and AgNPs. The structural, ultrastructural and thermal properties of the modified sealer were not affected by this modification, while demonstrating a perfect interfacial adaptation with the root canal dentine. As successful root canal therapy involves a combination of proper instrumentation, irrigation, obturation, and sealing, we suggest that our results are supportive of the near future potential of nanoparticles in clinical endodontics.
